# Q Fever Risk in Patients Treated with Chronic Antitumor Necrosis Factor-Alpha Therapy

**DOI:** 10.1155/2016/4586150

**Published:** 2016-08-30

**Authors:** Julianna Hirsch, Anna Astrahan, Majed Odeh, Nizar Elias, Itzhak Rosner, Doron Rimar, Lisa Kaly, Michael Rozenbaum, Nina Boulman, Gleb Slobodin

**Affiliations:** ^1^Bruce and Ruth Rappaport Faculty of Medicine, Technion, Haifa, Israel; ^2^Department of Internal Medicine A, Bnai Zion Medical Center, Haifa, Israel; ^3^Department of Rheumatology, Bnai Zion Medical Center, Haifa, Israel

## Abstract

Q fever is a zoonotic bacterial disease caused by* Coxiella burnetii*. Tumor necrosis factor-alpha (TNF-*α*) plays a pivotal role in the defense against infection with this Gram-negative coccobacillus. Theoretically, patients who are treated with anti-TNF-*α* medications are at risk for developing chronic Q fever. We present two patients who developed Q fever while being treated with anti-TNF-*α* agents and discuss the significance of timely diagnosis of* C. burnetii* infection in these patients.

## 1. Introduction

Q fever is a zoonotic bacterial disease caused by* Coxiella burnetii*. It spreads via inhalation or ingestion of contaminated air or food and occurs worldwide. Acute Q fever infection can manifest as a sudden febrile disease with cough, malaise, myalgia, headache, elevated liver enzymes, and thrombocytopenia. Meningitis, encephalitis, and myocarditis can also be seen in the course of primary infection. It is believed, however, that 20%–80% of cases of acute Q fever are asymptomatic. Progression towards chronic Q fever, present most often as endocarditis, vascular, or osteoarticular infection, can take place in some patients and be life threatening if not properly treated [1].

The diagnosis of Q fever should always be confirmed by serologic testing. During an acute attack, the antibody response to the* C. burnetii* phase II antigen predominates with the presence of IgM against phase I and phase II. Chronic disease is associated with rising titers of IgG against phase I antigen [2]. Males and individuals older than 40 years, particularly livestock handlers (including veterinarians, butchers, slaughterhouse workers, and farmers), are at greater risk of symptomatic disease, while patients with histories of heart valvular disease, endocarditis, or valvular implants as well as patients who are immune compromised may develop chronic infection more often [3].

*C. burnetii* functions as a strictly intracellular organism, affecting mononuclear phagocytes within which they multiply; survival of the bacteria is dependent on these phagocytic cells. Tumor necrosis factor-alpha (TNF-*α*) plays a crucial role in the defensive immune response to* C. burnetii* invasion and is responsible for early infection control [4].

We present herein two patients who developed Q fever while receiving chronic anti-TNF-*α* treatment.

## 2. Case Reports

### 2.1. Case 1

A 49-year-old male with a 12-year history of ankylosing spondylitis treated with infliximab at a dosage of 300 mg/kg for the last 4 years was referred for evaluation because of unexplained elevation of liver enzymes, disclosed by routine laboratory testing. The patient did not have specific complaints and denied fever, cough, malaise, or myalgias. He denied as well recent travel and use of medications other than infliximab nor alcohol and was not exposed to animals. The patient did not have known valvular heart disease, vascular aneurysms, or prostheses. No local outbreaks of any infectious disease, including Q fever, were reported at that time.

Physical examination of the patient revealed normal heart rate, arterial blood pressure, and body temperature and demonstrated limitation of lumbar and cervical spine mobility typical for ankylosing spondylitis. No jaundice, skin rash, or enlargement of lymph nodes, liver, or spleen was detected. Other physical findings including examination of heart and lungs were unremarkable.

Laboratory studies demonstrated elevated serum levels of aspartate transferase (AST) of 487 U/L (normal range 8–38 U/L), alanine transferase (ALT) of 1036 U/L (normal range 8–41 U/L), gamma-glutamyl transferase (GGT) of 90 U/L (normal range 11–50 U/L), alkaline phosphatase (ALP) of 74 U/L (normal range 40–129 U/L), and lactate dehydrogenase (LDH) of 564 U/L (normal range 240–480 U/L), while normal serum bilirubin level was normal. Synthetic liver function was normal, with serum albumin level of 4.79 g/dL (normal range 3.2–5.0 g/dL), total cholesterol level of 232 mg/dL (normal range 150–200 mg/dL), and INR of 1.16 (normal range 0.85–1.2). Serum C-reactive protein (CRP) was 2.4 mg/L; complete blood count and renal function were normal. Serologies for hepatitis A, B, and C viruses, Epstein-Barr virus (EBV), HIV, and cytomegalovirus (CMV) were negative. Anti-nuclear antibody (ANA) test was positive at 1 : 160 in a homogenous pattern, and anti-smooth muscle and anti-LKM antibodies were negative. Sonographic evaluation of the liver was unremarkable.

The patient's clinical condition remained stable for the next week, but his liver function tests did not improve. His scheduled infliximab infusion was placed on hold. Meanwhile, serology for Q fever performed by indirect immunofluorescence assay returned indicative for an acute infection with positive IgM against phase II and negative IgM against phase I and negative IgG antibodies against both phase I and phase II. PCR for* C. burnetii* DNA was not performed. Treatment with doxycycline 100 mg bid was administered for 10 days with rapid normalization of all liver enzymes. Serology for* C. burnetii* repeated 3 months later was negative for IgM and IgG antibodies. Treatment with infliximab was resumed at that time with no further complications.

### 2.2. Case 2

A 46-year-old female, suffering from skin psoriasis and treated with etanercept, 50 mg qw for the last two years, was admitted for evaluation because of systemic symptoms which had been developing progressively during the previous six months. Her complaints included generalized myalgia and weakness, constant headaches, diffuse abdominal pain, aphthous mouth ulcers, subfebrile temperatures up to 37.5°C, moderate night sweats, and 10 kg unintentional weight loss. She did not have any significant past medical history and denied the presence of valvular heart disease, vascular aneurysms, or prostheses.

Physical examination revealed two mouth ulcers, mild diffuse abdominal tenderness with no hepato- or splenomegaly, and was otherwise unremarkable. Laboratory tests showed mild leukopenia of 3.5 × 10^3^/mm^3^ with otherwise normal complete blood count and normal routine blood chemistry including liver and kidney function tests and CRP. Blood and urine cultures and serologic tests for EBV, CMV, HIV,* Brucella*, rheumatoid factor, and ANA were all negative. Computed tomography of chest and abdomen, total body technetium bone scan, and transthoracic echocardiography were normal. Gastroscopy revealed mild erosive gastritis, and colonoscopy was unremarkable. By the end of investigation, serologic tests for Q fever were performed by indirect immunofluorescence assay and returned positive with IgG titer against phase I >3200 and IgG titer against phase II = 3200, levels diagnostic for chronic Q fever. PCR for* C. burnetii* DNA was not performed. Treatment with etanercept was stopped, and transesophageal echocardiography was performed, which did not show valvular vegetations.

The patient started an 18-month long treatment with doxycycline, 100 mg bid, and hydroxychloroquine, 200 mg bid. Her physical condition improved significantly within two weeks of treatment. The titer of IgG against both phases I and II decreased to 800 after 6 months of treatment, and she is still asymptomatic on follow-up.

## 3. Discussion

Q fever is a global disease, caused by infection with* C. burnetii*, with cases reported sporadically or occasionally as outbreaks. While the bacterium largely affects persons in contact with livestock and its products, it can spread as well, although rare, by other means such as via arthropod vectors sexual contact or by blood transfusion. A particular subset of the population, namely, those who are treated by TNF-*α* inhibitors, might be at a disadvantage despite a paucity of reports on the prevalence and manifestations of Q fever in persons treated by anti-TNF-*α* medicines. The only study published on this topic compared seroprevalence of* C. burnetii* infection in patients with rheumatoid arthritis (RA) after the outbreak of Q fever in Netherlands [5]. The prevalence of past infection in RA patients, whether treated or nontreated with anti-TNF-*α* medicines, was similar, 15.8% and 13.8%, respectively. However, anti-TNF-*α* treated patients were more frequently diagnosed with chronic Q fever (7 of 57 seropositive patients, 12,3%), compared to anti-TNF-*α* naïve individuals (3 of 55 patients, 5.5%). This difference, while statistically nonsignificant in this study, can have relevance in clinical practice and be a reflection of poor control of early* C. burnetii* infection secondary to TNF-*α* inhibition [5]. Two recent case reports of progression of* C. burnetii* infection to chronic Q fever, as well as our patient of Case 2, should raise suspicion for infection with* C. burnetii* and the ensuing sickness with Q fever in patients under treatment with anti-TNF-*α* therapies and who are present with suspicious or unexplained systemic symptoms [6, 7]. Timely diagnosis of Q fever in these patients should lead, in our opinion, to immediate cessation of TNF-*α* inhibitor and administration of proper antibiotic treatment.

Indirect immunofluorescence assay and ELISA are the most widely used diagnostic tests nowadays, while PCR assays can be utilized as well for the early diagnosis of* C. burnetii* infection. Of importance, cross-reactions between* C. burnetii*,* Legionella*, and* Bartonella* antibodies have been described in the literature and should be considered in cases with nonclassical results of serologic testing, such as Case 1 with undetectable IgG antibodies on repeated examination.

Of importance, a monovalent, killed vaccine against* C. burnetii* is available nowadays and is designated for high-risk individuals including those who work with livestock [8]. Whether anti-Q fever vaccination of anti-TNF-*α* treated patients living in endemic areas can be of benefit needs to be examined.

In summary, infection with* C. burnetii* should be considered and specific serology testing performed in all patients being treated with anti-TNF-*α* therapies and suspicious for Q fever clinical presentation or with unexplained systemic symptoms.
